# Physiological response of *CmWRKY15-1* to chrysanthemum white rust based on TRV-VIGS

**DOI:** 10.3389/fpls.2023.1140596

**Published:** 2023-03-10

**Authors:** Qi Chen, Anchan Kuang, Haihong Wu, Di Liu, Xin Zhang, Hongyu Mao

**Affiliations:** ^1^ College of Forestry, Shenyang Agricultural University, Shenyang, China; ^2^ Institute of Flowers, Liaoning Academy of Agricultural Sciences, Shenyang, China

**Keywords:** chrysanthemum white rust, CmWRKY15-1, VIGS transient silence, antioxidant enzymes, defense related enzymes

## Abstract

Chrysanthemum White Rust (CWR) caused by *Puccinia horiana* Henn. is a major disease in the production process of chrysanthemum, which is widely spread all over the world and can be called “cancer” of chrysanthemum. To clarify the disease resistance function of disease resistance genes can provide a theoretical basis for the utilization and genetic improvement of chrysanthemum resistant varieties. In this study, the resistant cultivar ‘China Red’ was used as the experimental material. We constructed the silencing vector pTRV2-CmWRKY15-1 and obtained the silenced line named TRV-CmWRKY15-1. The results of enzyme activity after inoculation with pathogenic fungi showed that the activities of antioxidant enzymes SOD, POD, CAT and defense-related enzymes PAL and CHI in leaves were stimulated under the stress of *P*. *horiana*. In the WT, the activities of SOD, POD and CAT at the peak value were 1.99 times, 2.84 times and 1.39 times higher than that in TRV-CmWRKY15-1, respectively. And the activities of PALand CHI at the peak were 1.63 times and 1.12 times of TRV-CmWRKY15-1. The content of MDA and soluble sugar also confirmed that chrysanthemum was more susceptible to pathogenic fungi when *CmWRKY15-1* was silenced. The expression levels of *POD*, *SOD*, *PAL* and *CHI* at different time points showed that the expressions of defense enzyme related genes were inhibited in TRV-WRKY15-1 under the infection of *P. horiana*, which weakened the ability of chrysanthemum to resist white rust. In conclusion, *CmWRKY15-1* may increased the resistance of chrysanthemum to white rust by increasing the activity of protective enzyme system, which laid a foundation for breeding new varieties with disease resistance.

## 1 Introduction

Chrysanthemum is one of the most economical and popular flower crops with rich colors and patterns. CWR caused by *P. horiana* is one of the most important destructive diseases in the production process of chrysanthemum, which is widely spread around the world (Ye et al., 2005; Zeng et al., 2013; Bonde et al., 2015; Deenamo et al., 2018; Wang et al., 2020). The pathogens are obligate biotroph, which colonize young leaves and flower buds. They are easiest to germinate at 10-25 °C. The fungus produce only two spore stages, teliospores and basidiospores. Studies under the microscopes revealed that the telial pustules were found mostly on the lower side of the leaves (Zeng et al., 2013). During the period of infection with CWR, the pale-green to yellow white rust spots are found on the upper surface of the leaf. Centre of the spots turns brown and necrotic upon aging (Kumar et al., 2021). The disease caused degradation of flower quality and resulted in up to 100% yield loss, thus causing destructive damage (Bety and Pangestuti, 2021). At present, the research on chrysanthemum white rust is mostly focused on physical therapy and chemical therapy (Dheepa et al., 2016; Torres et al., 2017). Although biological fungicides and chemical fungicides can control the occurrence of CWR, *P. horiana* has developed resistance to various fungicides through gene mutation encoding the target protein of fungicide (Matsuzaki et al., 2021; Nuryani et al., 2021; Gavrilescu and Chisti, 2005). At the same time, excessive use of fungicides will cause serious environmental pollution. Therefore, breeding and popularizing disease-resistant varieties is the most economic, effective and environmental protection measure to control the occurrence of CWR (Bety and Pangestuti, 2021; Mekapogu et al., 2021). Looking for disease resistance genes and exploring disease resistance functions can provide theoretical basis for utilization of resistant chrysanthemum varieties and genetic improvement of varieties.

In the process of long-term co-evolution of plants and pathogens, many effective defense systems have been formed, in which the protective enzyme system plays a very important role. In plants, antioxidant enzymes (SOD, POD, CAT) and defense-related enzymes (PAL, CHI) are important components of the protective enzyme system (Liao et al., 2022). Wang found that the SOD, POD and PAL activities in immune specie increased rapidly after inoculation with *P. horiana*, however SOD, POD and PAL remained at a low and steady level in the highly susceptible one (Wang et al., 2020). WRKY is one of the largest families of transcriptional regulators in plants and is also an important regulator involved in signal transduction and transmission (Rushton et al., 2010). WRKY transcription factors not only participate in the process of plant seed development, but also can be induced to express in the response to various biological stress (Si et al., 2019). In this process, the activities of protective enzymes often changed. Wang found that compared with wild-type tobacco, the activities of SOD, POD and ascorbate peroxidase (ASP) in transgenic tobacco overexpressing *JsWRKY1* were significantly enhanced after inoculation with *Colletotrichum gloeosporioides* (Wang et al., 2016). Overexpression of *FvWRKY42* in *Arabidopsis thaliana* will increase the enzyme activities of SOD and CAT in transgenic lines and increase the resistance to powdery mildew (Wei et al., 2018). Overexpression of *GhWRKY39* in cotton significantly enhanced the activities of SOD,POD and CAT in transgenic lines, thus enhancing the defense against pathogens (Shi et al., 2014). In the early stage, we obtained the differentially expressed gene *CmWRKY15-1* through transcriptome sequencing (Dong et al., 2018). But, How does this gene change the physiological defense function in Chrysanthemum after inoculation with *P. horiana* is still unknown.

VIGS vector is a standard binary Ti plasmid derived vector, in which part of the virus genome is inserted for plant transformation mediated by *Agrobacterium tumefaciens*. VIGS vector is a recombinant virus, which can carry a segment of endogenous genes of the host. As a reverse genetics tool for studying plant gene function, VIGS is widely used because of its convenience (Gao et al., 2022). In this study, the resistant cultivar ‘China Red’ was used as experimental material. We obtained TRV-CmWRKY15-1 transient silenced lines using VIGS transient silencing technology. Under the condition of pathogen infection, the activity of related enzymes and the expression of resistance related genes were measured. Then we identified the physiological response of *CmWRKY15-1* to chrysanthemum white rust and analyzed the disease resistance function of *CmWRKY15-1*, which provides a theoretical basis for future chrysanthemum resistance breeding experiments.

## 2 Materials and methods

### 2.1 Plant materials and vectors

The white rust-resistant chrysanthemum cultivar ‘China Red’ was obtained from the laboratory of the Forestry College of Shenyang Agricultural University; the pTRV1 and pTRV2 plasmids were presented by Professor Zhu Pengfang of the Forestry College of Shenyang Agricultural University.

### 2.2 Reagents and culture medium

Infestation solution: 1 mmol · L^-1^ MgCl_2_, 10 mmol · L^-1^ MES, 200 μmol·L^-1^AS; Kanamycin: 50 mg · L^-1^; Rif: 50 mg · L^-1^; LB culture medium: tryptone 10 g, yeast extract 5 g, NaCl 5 g, distilled water to a constant volume of 1 L; YEP culture medium: tryptone 10g, yeast extract 10g, NaCl 5g, distilled water to a constant volume of 1 L.

### 2.3 pTRV2-CmWRKY15-1 instantaneous silencing system establishment of ‘China Red’

We selected a 216 bp fragment outside the conservative region as the silence fragment, based on the *CmWRKY15-1* reference sequence (GenBank: KC615369.1) and pTRV2 plasmid vector. We used the primer *CmWRKY15-1*-F/R (**Table 1**) for PCR amplification and recovered the product. EcoRI and BamHI were selected for double digestion of vector plasmid and recovered fragment. We used T_4_DNA ligase to connect the target fragment with the vector and transformed it into the competent state of Escherichia coli. We performed PCR validation on coliform solution and screened positive clones. The resulting plasmids were transformed into *Agrobacterium tumefaciens* strain EHA105 by freeze-thaw method.

**Table 1:** Primers used in this study.

Primer	Sequence (5′-3′)
*CmWRKY15* **-**1**-**F	CGGAATTCTTCTGATGAGGAGGACAGCGA
*CmWRKY15-*1*-*R	CGGGATCCGTTTCTTTGGGCTCGACTCGG
*TRV2-*F	GGGCTAACAGTGCTCTTGGTG
*TRV2-*R	CGGACCTCCACTCGCTGGAGG
*Actin-*F	TCCGTTGCCCTGAGGTTCT
*Actin-*R	GATTTCCTTGCTCATCCTGTCA
*PAL-F*	ATGGCACCGAAGCAAGTCACAC
*PAL-R*	GATACCCGAGTAACCCTGGAGGAG
*CHI-F*	GGAGGTGCCCATTGTAACAGGAAG
*CHI-F*	TCCCAACCCTTATCACACACTTTCTTC
*SOD-F*	TTAACCCTCTCAGCCGCCTCAG
*SOD-R*	AGTCCCTTTAAGCACAGCAACAGC
*POD-F*	CTGGAGGTCCTTCTTGGAAAGTGC
*POD-R*	GTCGGTTGGAGCAGGGATTTGAG

The underlined part is the restriction enzyme recognition site.

The *Agrobacterium tumefaciens* of pTRV1, pTRV2, pTRV2-CmWRKY15-1 were divided into LB solid medium containing kanamycin and rifampicin, and the medium was inverted into an incubator at 28 °C for dark culture for 2 days. We put the single colony into 10 mL LB liquid culture medium containing kanamycin and rifampicin. Cultures were incubated at 28°C incubator with shaking at 180 rpm for overnight until the bacterial solution is turbid. We took 1mL of the above liquid into 100mL YEP liquid medium, and used a 200 r · min^-1^, 28 °C shaking incubator to reproduce *Agrobacterium* until the concentration reached OD_600_≈0.8~1.0. After centrifugation at 6000 r·min^-1^ for 10 min, the pelleted bacteria were resuspended in infiltration buffer (10 mmol·L^-1^ MES, 1 mmol·L^-1^ MgCl_2_, 200 μmol·L^-1^ AS). Then we mixed pTRV1 bacterial solution with pTRV2、pTRV2-CmWRKY15-1 bacterial solution in a volume of 1:1, and placed them for 3 hours at room temperature. WT untreated was used as negative control group, pTRV1 and pTRV2 mixed bacterial solution was used as positive control group, and pTRV1 and pTRV2-CmWRKY15-1 mixed bacterial solution was used as experimental group.

We chose the 30 day old, 6-8 leaf stage, robust ‘China Red’ tissue culture seedlings, and soaked the whole plant in the control group and experimental group bacterial solution for 1h. There were 3 seedlings in each group, and a total of 8 groups were treated. Then we took out the plants and washed them with sterile water until there were no residual bacterial liquid on the surface. Next we planted them on MS culture medium, cultured them in dark at 25°C for 1 day, and then placed them in a tissue culture room at 25 °C, under a 16-h light/8-h dark photo-period for further culture.

### 2.4 Acquisition and identification of transgenic plants

In order to verify whether the gene was successfully silenced, we took samples three days after infection. We extracted total RNA from leaves of the ‘China Red’ using the RNA prep Pure Plant Kit (TIANGEN, Beijing), and synthesized first-strand cDNAs using the Prime Script™II 1^st^Strand cDNA Synthesis Kit following the manufacturer’s protocol (TAKARA, Japan). Then we used these cDNAs as templates, designed specific primers according to the coat protein gene sequence of TRV2 (registration number: Z36974.2) for PCR detection. At the same time, the gene expression of *CmWRKY15-1* in leaves was detected by qRT-PCR with primer *CmWRKY15-1*-RT-F/R using chrysanthemum Actin internal reference factor as control.

### 2.5 Verification of disease resistance function of *CmWRKY15-1*

We selected WT and silenced lines with good growth to inoculate *P*. *horiana*, the specific method of inoculation refer to the literature (Gao et al., 2021). Leaves of WT and silenced lines were sampled at 0, 24, 48, and 72 h after inoculation with *P*. *horiana*. Frozen leaf samples of WT and silenced lines of ‘China Red’ were used to measure the activity of defense enzymes, namely, SOD, POD, CAT, PAL and CHI. SODand POD activities were measured following the protocol by Sun (Sun et al., 2013). CHI, CAT and PAL activities were measured following the protocol by Kwon and Liu (Kwon & Nguyen, 2003; Liu et al., 2005). The content of MDA was determined following the method by Tsikas (Tsikas, 2017). The content of soluble sugar was determined by Dien (Dien et al., 2019). In addition, the relative expression levels of defense enzyme related genes *PAL*, *CHI*, *SOD* and *POD* were quantified by qRT-qPCR. All experiments were performed with three biological replicates. Primers are shown in **Table 1**.

### 2.6 Data processing and statistical analysis

The data obtained from the experiment were summarized by Excel 2013 and analyzed and verified by SPSS statistics analysis software.

## 3 Results

### 3.1 Construction of pTRV2-CmWRKY15-1 vector

**Figure 1:**
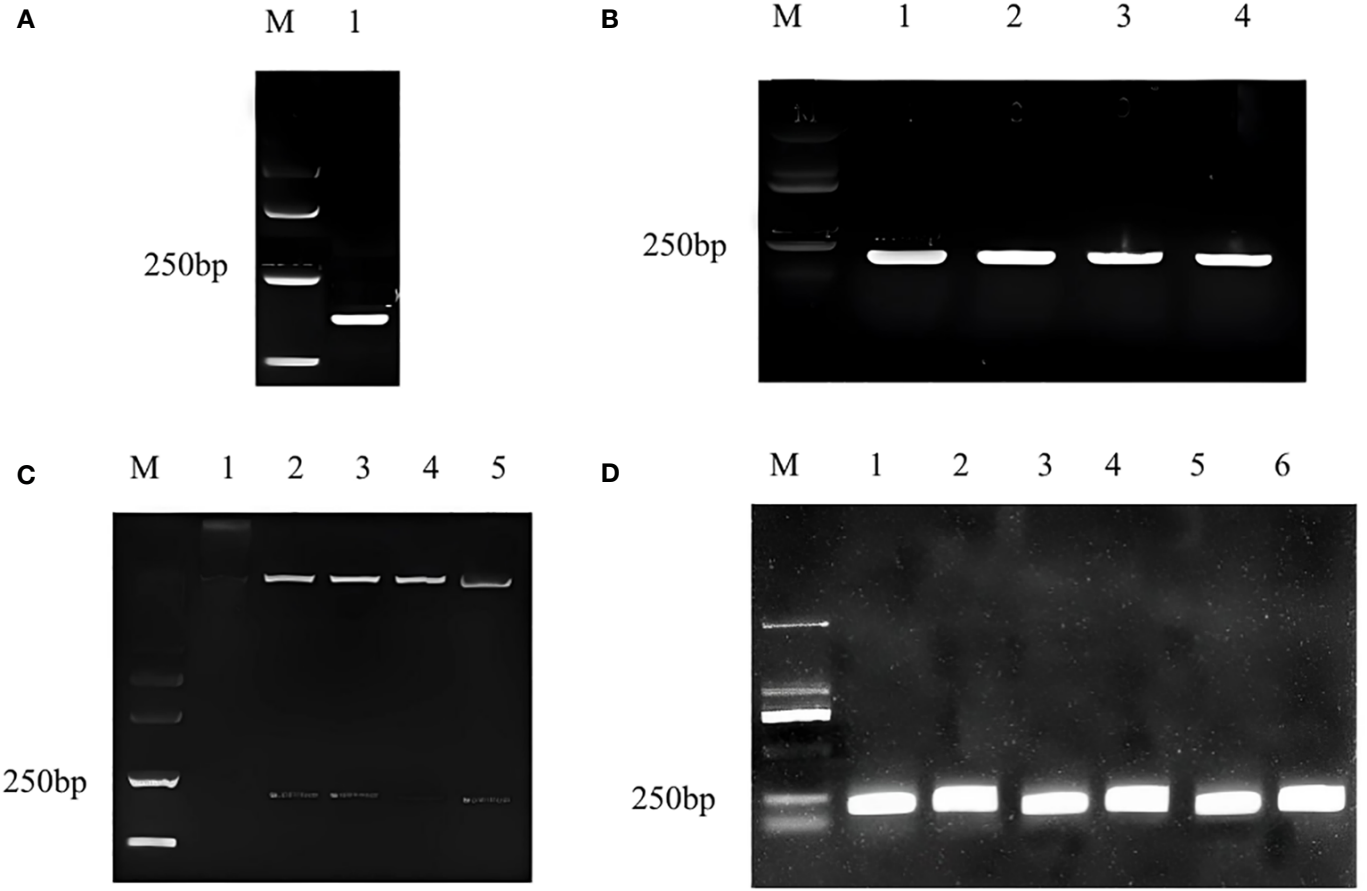
Construction of pTRV2-CmWRKY15-1 recombinant plasmid. **(A)** Target gene fragment: M, Marker; 1: Silent fragment of CmWRKY15-1. **(B)** Bacterial culture PCR for specific fragments: M, Marker; 1: First round PCR; 2: Second round PCR; 3: Third round control; 2: Second PCR;4:Fourth round PCR. **(C) **Double enzyme digestion verification: M, Marker; 1: positive round PCR; 3: Third round PCR; 4: Fourth round PCR; 5: Fifth round PCR. **(D)** Detection in Agrobactrtium bacterial liquid: M, Marker; 1: First round PCR; 2: Second round PCR; 3: Third round PCR; 4: Fourth round PCR; 5: Fifth round PCR; 6: Sixth round PCR.

The target fragment and pTRV2 vector were double digested with EcoRI and BamHI, and the 216 bp fragment (**Figure 1A**) conforming to the target band size of *CmWRKY15-1* and pTRV2 carrier large fragment were recovered respectively. We used T_4_DNA ligase to connect the recovered product overnight and transformed it into the escherichia coli. We performed PCR validation on coliform solution and the target band was appeared at 216 bp (**Figure 1B**). At the same time, double enzyme digestion was performed to verify that the target fragment and vector fragment were cut out, which proved that the silencing vector pTRV2-CmWRKY15-1 had been successfully constructed (**Figure 1C**). Then we transformed the recombinant plasmid into the *Agrobacterium tumefaciens* EHA105 by freeze-thaw method, and the corresponding target fragment appeared after the bacterial solution PCR verification (**Figure 1D**). Last, we mixed *Agrobacterium tumefaciens* solution with glycerol and stored at -80°C for standby.

### 3.2 Identification of instantaneous silenced transgenic plants

**Figure 2:**
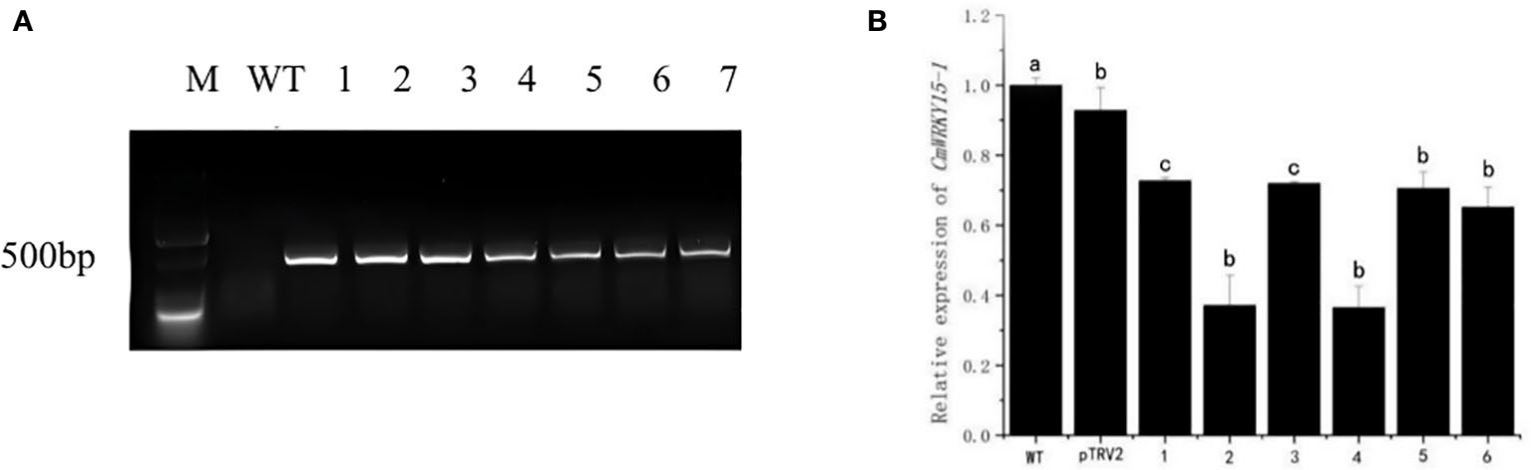
Identification of silenced chrysanthemum. **(A)** PCR detection: M, DNA marker; WT, Wild type; pTRV2, Positive control; 1-6, pTRV2-CmWRKY15-1 silenced plants. **(B)** qRT-PCR analysis of relative expression of CmWRKY15-1; WT, Wild type; pTRV2, Positive control; 1-6, pTRV2-CmWRKY15-1 silenced plants. Error bars show standard deviation of three replicates. Different small letters show significant difference (P<0.05).

We used the DNA of the silenced lines as the template, the DNA of WT as the negative control, and pTRV2 as the positive control, then we performed PCR detection with TPV2-F/R. As shown in the figure (**Figure 2A**), no target band was found in the WT plants, while the plants of the experimental group were able to amplify the target fragment which consistent with the length of pTRV2. This result indicated that pTRV2-CmWRKY15-1 had been successfully transferred into chrysanthemum ‘China Red’. The expression of *CmWRKY15-1* gene in WT (negative control), pTRV2 (positive control) and experimental leaves of ‘China Red’ showed that six transient silenced lines were obtained. The expression level of *CmWRKY15-1* gene in silenced lines were lower than that of WT and the expression amount were about 30% - 70% of WT (**Figure 2B**), indicating that *CmWRKY15-1* gene were effectively silenced in ‘China red’. The silenced line4 with the highest silenced efficiency was selected as the follow-up test material and named as the *CmWRKY15-1* silenced line (TRV-WRKY15-1).

### 3.3 Silencing of CmWRKY15-1 reduced the activity of protective enzymes in chrysanthemum under the infection of CWR

**Figure 3:**
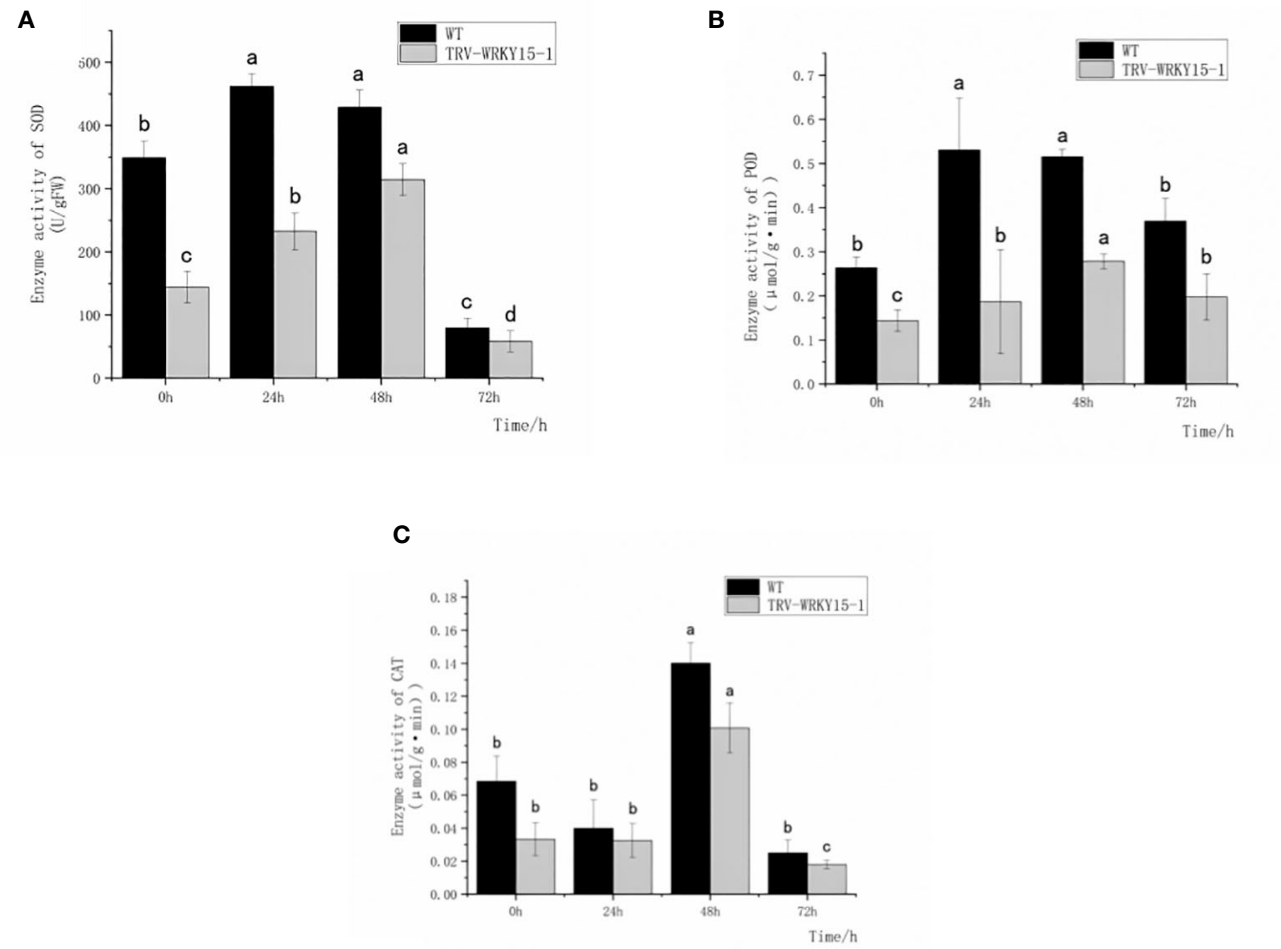
Determination of SOD, POD, CAT enzyme activity. **(A)** SOD activity. **(B)** POD activity. **(C)** CAT activity. Error bars show standard deviation of three replicates.The different small letters show significant difference (P<0.05).

In order to determine whether the silencing of *CmWRKY15-1* will affect the activities of protective enzymes in chrysanthemum under the infection of CWR, we measured the activities of antioxidant enzymes (SOD,POD,CAT) and defense-related enzymes (PAL,CHI) in the WT and TRV-WRKY15-1 at different time points. As shown in **Figure 3**, after inoculation the enzyme activities of SOD and POD in WT and TRV-WRKY15-1 all showed a trend of increasing first and then decreasing. The overall activity of SOD and POD in WT were higher than that in TRV-WRKY15-1 (**Figures 3A, B** **)**. The enzyme activities of SOD and POD of WT reached the peak value at 24 h after inoculation, which were 2.06 times and 2.84 times of the TRV-WRKY15-1. However, the TRV-WRKY15-1 reached the peak value of enzyme activity at 48h, which were 0.73 times and 0.54 times of the WT (**Figures 3A, B** **)**. It can be seen that compared with WT, TRV-WRKY15-1 began to respond to pathogen stress later. After inoculation, the activity of CAT in WT and TRV-WRKY15-1 showed a trend of decreasing first and then increasing. Although the activity of CAT in the WT and TRV-WRKY15-1 reached the peak value at 48 h after inoculation, the enzyme activity of TRV-WRKY15-1 was 0.71 times lower than that of the WT (**Figure 3C**). From the above data, it can be seen that pathogen stress can stimulate the antioxidant enzyme activity in chrysanthemum. Compared with WT, TRV-WRKY15-1 began to respond to pathogen stress later. The silencing of *CmWRKY15-1* reduced the enzyme activity in general and weakened the disease resistance of chrysanthemum ‘China Red’.

**Figure 4:**
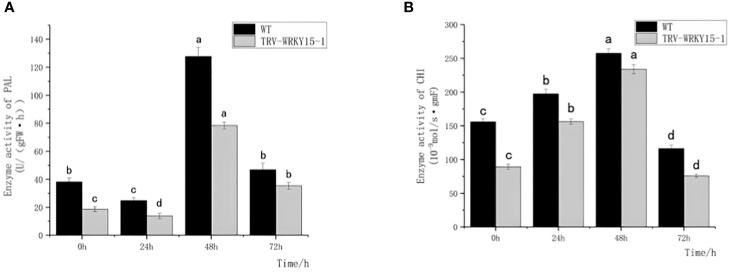
Determination of PAL,CHI enzyme activity. **(A)** PAL activity. **(B)** CHI activity. Error bars show standard deviation of three replicates. The different small letters show significant difference (P<0.05).

PAL and CHI, as key enzymes of defense reaction, play very important roles in enhancing plant disease resistance. As shown in **Figure 4**, the overall activities of PAL and CHI in WT were higher than that in TRV-WRKY15-1. Although the PAL and CHI enzyme activities in WT and TRV-WRKY15-1 reached their peak values at 48 h, the PAL and CHI enzyme activities were only 0.61 times and 0.81 times of those in WT (**Figures 4A, B** **)**. It can be seen the silencing of *CmWRKY15-1* weakened the defense ability of chrysanthemum, thereby reduced the resistance to white rust.

**Figure 5:**
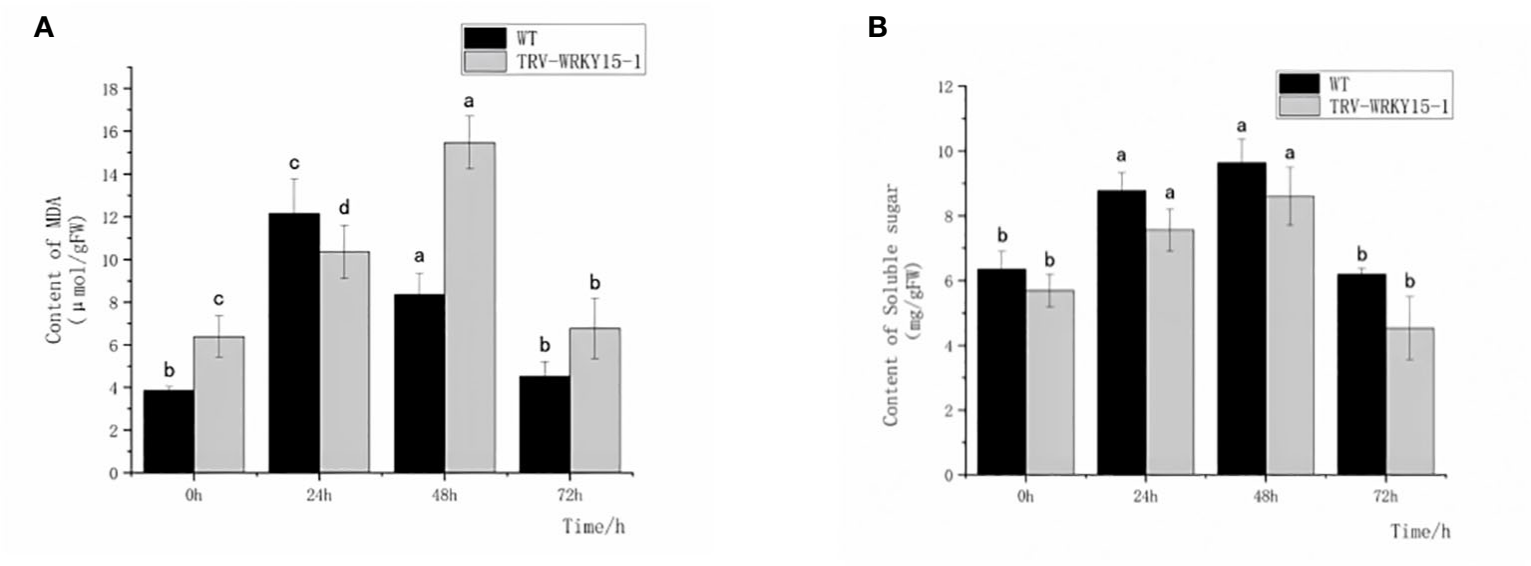
Determination of MDA and Soluble sugar content. **(A)** MDA content. **(B)** Soluble suger content. Error bars show standard deviation of three replicates. The different small letters show significant difference (P<0.05).

In order to explore the damage degree of WT and TRV-WRKY15-1 at different time points after inoculation, we also measured the content of MDA and soluble sugar. The content of MDA, an osmotic regulator, can directly reflect the degree of plant damage. The accumulation of soluble sugar content can supplement the required nutrients, reducing osmotic potential and maintaining cell osmotic pressure. As shown in **Figure 5**, after inoculation, the MDA content in WT and TRV-WRKY15-1 showed a trend of increasing first and then decreasing. Except for 24 h, the MDA content in TRV-WRKY15-1 was higher than in WT and reached its peak at 48 h, about 1.85 times of the WT. These indicated that the disease resistance of the TRV-WRKY15-1 was lower than the WT (**Figure 5A**). It showed that the TRV-WRKY15-1 was more seriously damaged and more vulnerable to damage caused by pathogens infection. However, the soluble sugar content in TRV-WRKY15-1 was slightly lower than the WT, and the overall change trend was first increased and then gradually decreased (**Figure 5B**). These indicated that the silencing of *CmWRKY15-1* weakened the plant’s ability to provide nutrition and energy, decreased the metabolic energy, thus weakened the defense against pathogens.

### 3.4 Silencing of CmWRKY15-1 reduced the expression of defense-related genes in chrysanthemum under the infection of CWR

**Figure 6:**
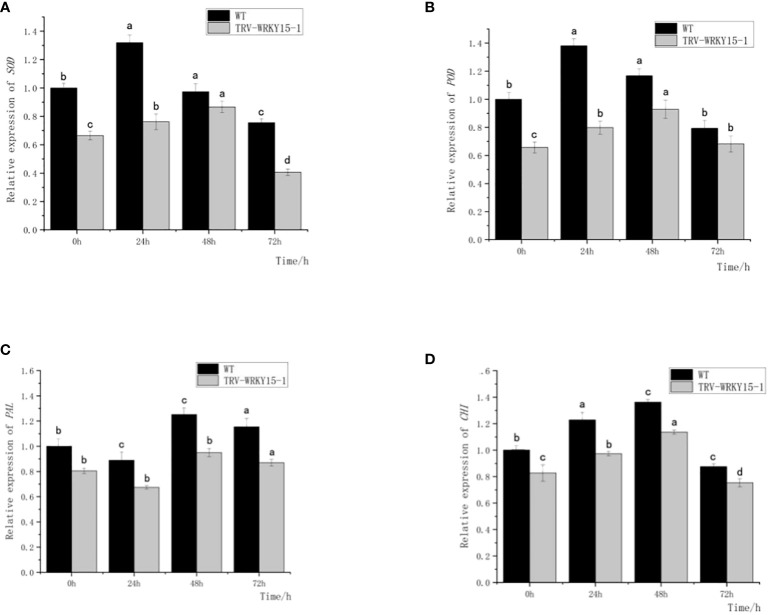
Expression of SOD, POD, PAL, CHI genes. **(A)** Transcript levels of SOD gene. **(B)** Transcript levels of POD gene. **(C)** Transcript levels of PAL gene. **(D)** Transcript levels of CHI gene. Error bars show standard deviation of three replicates.The different small letters show significant difference (P<0.05).

As shown in **Figure 6**, the expression of *SOD* and *POD* genes in both WT and TRV-WRKY15-1 showed a trend of first increasing and then gradually decreasing after inoculation. Both of them reached the peak at 24 h in WT, about 1.32 times and 1.38 times of that not inoculated (**Figures 6A, B** **)**. In TRV-WRKY15-1, the expressions of *SOD* and *POD* genes reached the maximum value at 48 h, about 1.30 times and 1.41 times of that not inoculated, and 0.89 times and 0.79 times of WT at the same time. The overall expression of *SOD* and *POD* genes in TRV-WRKY15-1 were lower than that in WT (**Figures 6A, B** **)**. The change trend of *CHI* in WT and TRV-WRKY15-1 after inoculation were similar (**Figure 6C**), and showed a trend of first increasing and then gradually decreasing. The maximum expression all appeared at 48h, and reached 1.36 times and 1.37 times of that not inoculated, respectively. At the same time, the expression in TRV-WRKY15-1 was 0.83 times of WT (**Figure 6C**). As shown in **Figure 6D**, the expression of *PAL* in WT and TRV-WRKY15-1 showed a downward-upward-downward trend after inoculation, and all reached the peak at 48 h, about 1.25 times and 1.18 times of that not inoculated. At this time, the expression of *PAL* in TRV-WRKY15-1 was 0.76 times of WT. It can be inferred from the above data that the silencing of *CmWRKY15-1* weakened the expression of defense-related genes PAL, CHI, SOD and POD, which was consistent with the results of enzyme activity measurement. These results proved that the silencing of *CmWRKT15-1* weakened the activity of protective enzymes in chrysanthemum and reduced the resistance of chrysanthemum to white rust.

## 4 Discussion

Chrysanthemum morifolium are used for cut flowers and potted plants in commercial production regions of the world. Most of the cut flowers of chrysanthemum produced in China will be exported to Japan, South Korea and other countries. Once the quality of cut chrysanthemum is damaged, the export efficiency would be greatly reduced. Preventing flowers from infecting disease is the most effective way to protect the quality of flowers. CWR is easy to occur in the environment of low temperature and high humidity (Yoo and Roh, 2014). The occurrence of CWR reduces the commercial value of chrysanthemum, leading to significant economic losses in the cut flower industry. The pathogen spores are easy to spread by the wind in autumn and winter. Then it quickly infects the leaf surface which brings great difficulties to its prevention and control. Breeding and using resistant varieties is the most economic, effective and environmentally safe measure for disease control (Bety and Pangestuti, 2021; Mekapogu et al., 2021). Breeding new disease resistant varieties through transgenic technology will become an effective way to resist CWR (Gao et al., 2022). In this study, the silencing vector was constructed and successfully transferred to chrysanthemum ‘China Red’. The relative expression of *CmWRKY15-1* in TRV-WRKY15-1 lines was 0.3~0.7 times of the WT. The TRV-WRKY15-1 lines could be used as an important resource for disease resistance function analysis of *CmWRKY15-1* in the late stage.

SOD, POD and CAT are important antioxidant enzymes in plants. They can reduce or block the damage of reactive oxygen free radicals to plant tissues (Koramutla et al., 2014), and enhance the tolerance of plants to biologic stress and abiotic stress. Xiang found that *AtWRKY70* positively regulates the response of *Arabidopsis thaliana* to biological stress by strengthening the antioxidant enzyme system, maintaining the stability of membrane lipids (Xiang et al., 2021).In apples, when *MdWRKY40* was silenced, the enzyme activities of SOD and CAT were significantly enhanced after inoculation with *Powdery mildew*, and the expression of related genes *SOD* and *CAT* were also significantly up-regulated, thus improving the basic resistance of apple plants to powdery mildew (Sha et al., 2021). In addition, excessive accumulation of ROS will also lead to lipid peroxidation inthe biofilm and the accumulation of MDA, which can directly affect the damage degree of plants. Some studies have shown that when rice was inoculated with *Magnaporthe grisea*, the content of MDA was significantly increased (Hu et al., 2017). In this study, we founded that the SOD, POD and CAT activities of WT were significantly higher than those of TRV-WRKY15-1 within 24~72 h after inoculation.We speculated that the silencing of *CmWRKY15-1* delayed the response of antioxidant enzymes to the infection of pathogens. Previous researches also showed that the activity of POD was linked to the cell wall strengthening (Sasaki et al., 2004; Grabber, 2005), in susceptible species, activity of peroxidase enzyme was inhibited (Peters et al., 2017). Here, the lower activity of POD in TRV-WRKY15-1 than WT might also contribute to the cell wall weakening which in turn promotes the pathogen penetration. However, the activity of CAT in WT and TRV-WRKY15-1 were significantly decreased at 24 h, which may be due to the fact that the H_2_O_2_ in the plants were still at a safe level. The CAT activity rose sharply at 48 h indicating that the accumulation of H_2_O_2_ have exceeded the balance state *in vivo* at this time. Some studies showed that the activity of antioxidant enzymes did not always increase, the activities gradually decreased with the extension of stress time (Jing et al., 2013). Therefore, in this study, the activities of SOD, POD and CAT of WT and TRV-WRKY15-1 began to decrease in the later stage. In addition, the content of MDA in the WT and TRV-WRKY15-1 were significantly increased at 24 h, which proved that the harm is more serious at this time.

PAL and CHI have been suggested to be involved in plant defense responses against fungal infection (Zhao et al., 2008). PAL is the key enzyme of shikimic acid pathway in plants, and plays a key role in the accumulation of lignin and the synthesis of phytoalexins and phenols. Chitinase can hydrolyze the chitin in the cell wall of the mycelium, thereby destroying the mycelium and inhibiting the growth of the mycelium (Zhang et al., 2020). When the kiwifruit was inoculated with *P. expansum*, the activies of PAL and CHI increased significantly (Cao et al., 2022). After the lily was infected by *Fusarium oxysporum*, the PAL and CHI activities showed a trend of increasing first and then decreasing (Zhang et al., 2020). In this study, the activities of CHI increased significantly at 24 h-72 h after inoculation, indicating that the plant’s defense system was stimulated at this time. But the activity of PAL was decreased at 24 h then rose rapidly at 48 h, this result is not consistent with that of other species. We speculated that different species might employ different defensive strategies to cope with pathogen infection. In addition, compared with WT, TRV-CmWRKY15-1 showed lower enzyme activity at any time points, indicating that the silencing of *CmWRKY15-1* weakened the defense ability of plants and weakened the resistance of CWR.

In order to further verified the conclusion, we also measured the expression of the genes encoded by the protective enzymes. The results showed that the expressions of *SOD*, *POD*, *PAL* and *CHI* in both WT and TRV-CmWRKY15-1were up-regulated first and gradually decreased with the extension of inoculation time. In addition, it can be seen that the expressions of TRV-CmWRKY15-1 were lower than that of the WT at any time points. These results were consistent with the results of enzyme activity measurement. In a word, the silencing of *CmWRKY15-1* reduced the activity of protective enzymes in chrysanthemum, and *CmWRKY15-1* has a positive regulatory effect on chrysanthemum white rust.This result lays a theoretical basis for the cultivation of new chrysanthemum varieties with disease resistance.

## Data availability statement

The datasets presented in this study can be found in online repositories. The names of the repository/repositories and accession number(s) can be found in the article/supplementary material.

## Author contributions

HM and QC designed the study and wrote the manuscript. QC and AK performed most of the experiments. HW, DL and XZ analyzed the data and discussed the article. All authors contributed to the article and approved the submitted version.
